# Strain-specific spleen remodelling in *Plasmodium yoelii* infections in Balb/c mice facilitates adherence and spleen macrophage-clearance escape

**DOI:** 10.1111/j.1462-5822.2010.01523.x

**Published:** 2011-01

**Authors:** Lorena Martin-Jaular, Mireia Ferrer, Maria Calvo, Anna Rosanas-Urgell, Susana Kalko, Stefanie Graewe, Guadalupe Soria, Núria Cortadellas, Jaume Ordi, Anna Planas, James Burns, Volker Heussler, Hernando A del Portillo

**Affiliations:** 1 Barcelona Centre for International Health ResearchBarcelona, Spain; 2 Unitat de Microscòpia Confocal, Serveis Cientificotècnics, Facultat de Medicina, Universitat de Barcelona- IDIBAPSBarcelona, Spain; 3 Bioinformatics Unit, IDIBAPS, Hospital ClinicBarcelona, Spain; 4 Bernhard Nocht Institute for Tropical Medicine, Department of Molecular Parasitology, Malaria laboratoryHamburg, Germany; 5 Department of Brain Ischemia and Neurodegeneration, Institut d'Investigacions Biomèdiques de Barcelona (IIBB)-Consejo Superior de Investigaciones Científicas (CSIC), Institut d'Investigacions Biomèdiques August Pi i Sunyer (IDIBAPS)Barcelona, Spain; 6 Unitat de Ressonància Magnètica Experimental, Plataforma d'Imatge, IDIBAPSBarcelona, Spain; 7 Unitat de Microscòpia Electrònica, Serveis Cientificotècnics, Facultat de Medicina, Universitat de Barcelona- IDIBAPSBarcelona, Spain; 8 Department of Anatomical Pathology, Hospital ClinicBarcelona, Spain; 9 Department of Microbiology and Immunology, Drexell University Medical CollegeQueen Lane, PA, USA; 10 Institució Catalana de Recerca i Estudis Avançats (ICREA)Barcelona, Spain

## Abstract

Knowledge of the dynamic features of the processes driven by malaria parasites in the spleen is lacking. To gain insight into the function and structure of the spleen in malaria, we have implemented intravital microscopy and magnetic resonance imaging of the mouse spleen in experimental infections with non-lethal (17X) and lethal (17XL) *Plasmodium yoelii* strains. Noticeably, there was higher parasite accumulation, reduced motility, loss of directionality, increased residence time and altered magnetic resonance only in the spleens of mice infected with 17X. Moreover, these differences were associated with the formation of a strain-specific induced spleen tissue barrier of fibroblastic origin, with red pulp macrophage-clearance evasion and with adherence of infected red blood cells to this barrier. Our data suggest that in this reticulocyte-prone non-lethal rodent malaria model, passage through the spleen is different from what is known in other *Plasmodium* species and open new avenues for functional/structural studies of this lymphoid organ in malaria.

## Introduction

The spleen is a complex organ that is perfectly adapted to selectively filtering and destroying senescent red blood cells (RBCs), infectious microorganisms and *Plasmodium*-infected RBCs (pRBCs) (Bowdler, 2002). Such filtering capacity is related to the complex structure of the spleen as it consists of a trabecular complex structure formed by: (i) the white pulp, lymphoid tissue containing the majority of immune effector cells, (ii) the red pulp, a reticular meshwork where destruction of senescent, aberrant RBCs and pRBCs occurs and (iii) a marginal zone lying between the white pulp and the red pulp, where inert particles, bacteria and viruses are eliminated. In addition, blood enters the spleen through a central artery that branches into capillaries, most of which empty into the filtration beds of the red pulp before reaching the venous system in a so-called open system. The spleen is therefore a complex organ whose 3D structure consists of distinct microanatomical zones exquisitely adapted to performing different functions.

The *Plasmodium yoelii* rodent model has been extensively used to study molecular aspects of virulence inmalaria mainly because of the existence of strains with different cellular tropisms, growth curves and clinical outcomes, such as the reticulocyte-prone non-lethal *P. yoelii* 17X strain and the normocyte-prone lethal *P. yoelii* 17XL strain (Pattaradilokrat *et al*., 2008). Noticeably, in experimental infections of Balb/c mice with the *P. yoelii* 17X strain, the open circulation of the spleen is temporarily changed to a closed circulation with the formation of syncytial layers of fibroblasts that form physical barriers, termed barrier cells (Weiss *et al*., 1986). Closing of the circulation was demonstrated by the absence of carbon particles in red pulp filtration beds following injection of infected mice during precrisis (a period of increasing parasitaemia). Precrisis was also characterized by intense spleen erythropoiesis and it was thus proposed that barriers cells protect immature RBCs from destruction by parasites before maturation and release into circulation to compensate for the anaemia caused by these experimental infections. In striking contrast, barrier cell-dependent remodelling did not occur in infections with the *P. yoelii* 17XL strain in Balb/c mice (Weiss *et al*., 1986).

Several promising methodologies have been developed enabling intravital and magnetic resonance imaging (MRI) of cells within their *in vivo* tissue environment in malaria (Amino *et al*., 2006; Heussler and Doerig, 2006; Sturm *et al*., 2006; Penet *et al*., 2007). We have implemented these techniques to address the dynamic features of the passage of *P. yoelii* 17X and 17XL strains through the spleen of Balb/c mice and have revisited global transcriptional analysis and histopathology of this organ in these experimental infections. Our data demonstrate that *P. yoelii* 17X induces a spleen blood barrier of fibroblastic origin to which infected reticulocytes adhere facilitating macrophage-clearance escape.

## Results

### Construction and characterization of *P. yoelii* 17X and 17XL clonal lines expressing green fluorescent protein (GFP)

To implement intravital imaging, transgenic lines of the *P. yoelii* 17X and 17XL strains expressing the mutant 3 variant of GFP from *Aequorea victoria* were constructed (JB), using the vector and conditions previously described in *Plasmodium berghei* (Franke-Fayard *et al*., 2004). Analysis of GFP transgenic parasites revealed that GFP expression did not alter growth curves and tropism when compared with the original strains (Fig. S1). Clonal transgenic lines from 17X and 17XL were obtained through standard methodologies and used throughout this study.

### Red pulp from spleens of animals infected with 17X contains a significantly higher number of parasites than red pulp from animals infected with 17XL

**Fig. 1:**
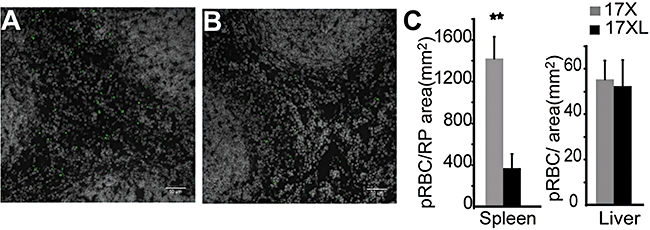
Increased retention of *P. yoelii* 17X GFP parasites in the red pulp of the spleen. A, B. Wide-field fluorescence microscopy images of GFP transgenic parasites (green) and nucleic acids (gray) on immunostained cryosections of the spleen of mice infected with 17X (A) or 17XL (B) parasites on day 3 p.i., corresponding to 1% peripheral parasitaemia. pRBCs accumulate in the red pulp, the area with less density of nuclei. Scale bars represent 50 µm. C. Quantification of parasite retention in the red pulp of the spleen and the liver in selected areas of 0.2 mm^2^ and 0.4 mm^2^, respectively, expressed as number of particles per area (pRBC mm^−2^). Values are the mean ± SEM of four mice (analysed with Student's *t*-test). Differences are statistically significant (**P < 0.01).

To investigate early spleen events (3–4 days post infection, p.i.) following the induction but not the complete formation of a spleen blood barrier of fibroblastic origin (Weiss *et al*., 1986), when no confounding effects are observed as a result of the complexity of infection in this model (Table 1), we performed immunohistofluorescence analysis of cryosections of spleens stained with anti-GFP antibody. Noticeably, quantification of GFP parasites in the red pulp of the spleen showed a 3.8-fold increase in 17X parasites compared with 17XL parasites on day 3 p.i. (Fig. 1A–C). Comparable results were obtained in cryosections of spleens stained with Giemsa and visualized under polarized light microscopy, which detects parasites because of the refringent properties of haemozoin (Fig. S2). Similar quantification of 17X and 17XL parasites in cryosections of the liver (Fig. 1C) revealed no differences in numbers, indicating that the higher 17X parasite retention was specific to the red pulp of the spleen.

**Table 1:** Balb/c – GFP *P. yoelii* 17X *and* 17XL murine malaria model.

Day p.i.	3				4			
		
Line	L-GFP	L	NL-GFP	NL	L-GFP	L	NL-GFP	NL
Parasitaemia (%)	1.1 ± 0.3	1.1 ± 0.2	1.1 ± 0.2	1.1 ± 0.2	9.9 ± 1.4	10.3 ± 0.4	9.2 ± 0.6	9.8 ± 0.2
Reticulocytemia (%)	2.7 ± 0.4	2.9 ± 0.7	2.5 ± 0.4	2.8 ± 0.5	2.3 ± 0.3	2.4 ± 0.5	2.9 ± 0.5	2.7 ± 0.3
pRet/pRBCs (%)	60.0 ± 9.1	62.8 ± 4.6	69.2 ± 9.5	72.7 ± 5.8	23.4 ± 5.1	24.7 ± 4.0	32.2 ± 10.0	38.0 ± 14.6
ring/pRBCs (%)	31.4		30.8		39.2		31.8	
troph/pRBCs (%)	64.7		62.7		57.3		63.5	
sch/pRBCs (%)	3.9		6.7		3.4		4.8	
S. w. (g)	0.1 ± 0.0	0.1 ± 0.0	0.1 ± 0.0	0.1 ± 0.0	0.3 ± 0.0	0.3 ± 0.0	0.3 ± 0.0	2.2 ± 0.0
RP/WP	2.3 ± 0.3		2.0 ± 0.2		0.8 ± 0.2		0.8 ± 0.1	

Different parameters were calculated in Balb/c mice infected with wild-type and transgenic parasites on days 3 and 4 p.i. when all experiments were done. Parasitaemias were calculated in groups of 12 animals. Reticulocytemia and percentage of parasitized reticulocytes (pRet) were measured in at least six mice per group. Percentages of asexual forms [rings, trophozoites (troph) and schizonts (sch)] were assessed in four mice per group counting at least 250 pRBCs per animal. Spleen weight (S.w.) and ratio of red pulp to white pulp area (RP/WP) were measured in 3–4 animals per group. The results are expressed as mean ± SEM. Statistical analysis was used to compare differences between the lethal and non-lethal group on those days, with no significant differences found.

### Differences in parasite load in the red pulp of the spleen cannot be explained by macrophage activity

**Fig. 2:**
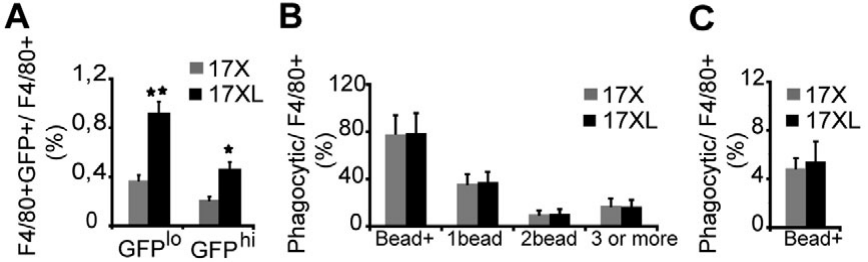
Flow cytometry analysis of red pulp macrophage activity on day 4 p.i. A. Splenocytes of mice infected with 17X and 17XL GFP transgenic parasites were labelled with an Alexa Fluor 647 conjugated antibody against F4/80 and analysed in a FacsCANTO flow cytometer. The gate was set to exclude debris and pRBCs. Percentages of F4/80^-^/GFP^+^ cells were 1.75 ± 0.26 for 17XL and 1.62 ± 0.29 in the 17X. No statistically significant differences were found between the two groups (*n* = 7 per group) (Student's *t*-test). F4/80^+^GFP^+^ cells were gated into two populations according to differences in GFP intensity. The mean ± SEM of GFP fluorescence of GFP^lo^/GFP^hi^ populations was 1480.39 ± 36.52/13 961.70 ± 66 for 17X and 1503.86 ± 48.17/12 730.85 ± 407.79 for 17XL. Quantification of the percentage of F4/80^+^GFP^lo^ and F4/80^+^GFP^hi^ cells is expressed as the mean ± SEM of six independent experiments (analysed with Student's *t*-test). Differences are statistically significant (*P < 0.05, **P < 0.01). B, C. Phagocytic activity measurements using fluorescent beads in F4/80^+^ spleen cells of mice infected with 17XL and 17X wild-type parasites. B. The percentage of macrophages obtained from infected spleens that had ingested *in vitro* one, two or three or more beads was calculated as a function of F4/80^+^ cells. After 1 h, around 78% of macrophages in both groups had ingested the fluorescent beads. C. Percentage of F4/80 cells that had incorporated fluorescent beads after *in vivo* i.v. injection. B, C. Mean ± SEM is shown (*n* = 6–7). No significant differences were observed between the lethal and non-lethal groups.

The differences observed in the accumulation of parasites in the red pulp of the spleen during experimental infections with *P. yoelii* 17X and 17XL could be due to differences in parasite engulfment by macrophages or other phagocytic cells. In order to test this hypothesis, Balb/c mice were infected with the *P. yoelii* GFP transgenic lines. Thereafter, splenocytes were stained with F4/80 antibody specific for red pulp macrophages and analysed by flow cytometry (Fig. 2A). The number of macrophages containing parasites was calculated as the percentage of F4/80^+^GFP^+^ cells as a function of F4/80^+^ cells. As we could not determine numbers of double-positive cells on day 3 p.i. as a result of the scarcity of F4/80^+^GFP^+^ cells, we performed the test on day 4 p.i., when there were still higher numbers of non-lethal parasites in the spleens of infected mice (13.83 ± 1.64% for 17X and 9.90 ± 0.19% for 17XL; *P* = 0.0160). Phenotypic analysis of splenocytes revealed higher proportions of F4/80^+^GFP^+^ cells in animals infected with 17XL than in those infected with 17X (Fig. 2A), even though the non-lethal strain had higher numbers of parasites and F4/80^+^ macrophages in infections (12.5 ± 1.99% of spleen cells for 17X and 6.64 ± 1.46% of spleen cells for 17XL; *P* = 0.0452). Of note, there was a pool of unidentified F4/80^-^GFP^+^ phagocytic cells in both parasite lines; because there were no significant differences in numbers with respect to total spleen cells, we did not investigate these further.

To evaluate the possibility that the differences detected might have been due to distinct phagocytic capacities of F4/80 cells, we determined the uptake of fluorescent beads by these cells. We demonstrated that the phagocytic capacity of spleen macrophages obtained from infections with 17X and 17XL was not significantly different either *in vitro* (Fig. 2C) or *in vivo* (Fig. 2D). These results exclude differences in engulfment by macrophages as the cause of the higher numbers of 17X parasites observed in the red pulp of the spleen.

### Spleen closure and spleen blood flow are similar in infections with 17X and 17XL

**Fig. 3:**
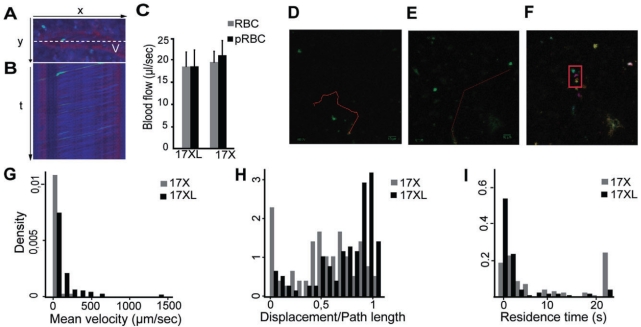
Intravital imaging of GFP parasites in the spleen. A, B, C. Spleen blood flow measurements. Representation of xy image (A) and xt image (B) from a line-scan of the central lumen of the same vessel (white line). Spleen vessel (V) showing plasma with 70 kDa dextran (red), pRBC (green) and erythrocyte reflection (blue). C. Volumetric blood flow quantification from pRBCs and surrounding RBCs in spleens infected with both lines. Data are expressed as the mean ± SEM (µl s^−1^) of three mice infected with each line at 1% parasitaemia (day 3 p.i.), considering vessels of different sizes (6–17 µm diameter) at different phases of the cardiac cycle. No significant differences were observed. D, E, F. Images representing maximum intensity projection of five different depths (Z) at one time point. Red line represents 4D manual tracking of particles of 17X (11.8 s) (D) and 17XL (2.2 s) (E) GFP parasites using MTrackJ. Tracking was performed with the depth information from Z-coded colour stacks (F). Depth code: yellow (0 µm), orange (2 µm), pink (4 µm), blue (6 µm), green (8 µm). The box shows movement in z-axis of the same particle. Distribution of GFP particles by mean velocity (G), directionality (H) and residence time (I). Data correspond to 120 particles of each line from three independent experiments analysed with the equality-of-medians test. The 17X/17XL medians are 22.58/103.95 µm s^−1^ (G), 0.53/0.75 (H) and 4.61/0.67 s (I). Differences between the two lines in (G) (H) and (I) are statistically significant (*P* < 0.001). The location of the particles quantified is comparable in both lines: 52.5% of 17X and 51.67% of 17XL outside vessels, 17.5% of 17X and 15.83% of 17XL in vessels with a diameter < 10 µm and 29.17% of 17X and 32.5% of 17XL in vessels with a diameter > 10 µm.

Differences in spleen closure and blood flow could have also been responsible for differential input of pRBCs to the red pulp and for altering the chances of interaction with spleen cells. To determine whether the higher retention of parasites in the spleen of Balb/c mice infected with 17X was due to differences in spleen closure, we analysed the uptake capacity *in vivo* of 3 µm fluorescent beads. No differences were observed in the trapping capacity of the spleen of mice infected with either 17X or 17XL parasites at this early time p.i. (Fig. S3). To discard differences in spleen blood flow, we implemented intravital imaging of the spleen in malaria. Thus, mice were anaesthetized and their spleens exposed and visualized under a high-speed multiphoton confocal microscope fitted with an inverted 63x glycerol objective (Movie S1). Intravital microscopy of spleen vessels with different diameters (6–17 µm) was optimized to perform a line-scan image of the centre of the vessels (Fig. 3A and B). Volumetric blood flow was calculated from pRBC and RBC velocity based on the slope of fluorescent streaks (Zhong *et al*., 2008). No differences in spleen blood flow were observed between 17X- and 17XL-infected mice (Fig. 3C). In addition, the values were not significantly different from spleen blood flow in uninfected mice injected with FITC-labelled RBCs (28.4 ± 4.5 µl s^−1^, anova*P* = 0.2625).

### Intravital imaging of the spleen revealed adherence of the *P. yoelii* 17X line

Having excluded differences in engulfment by macrophages, spleen closure and spleen blood flow as reasonable explanations for the significantly higher numbers of 17X than 17XL parasites observed in the red pulp of the spleen, we aimed to characterize the dynamic behaviour of these parasites and to quantify their mobility within the spleen. These parameters have been recently used to demonstrate *in vivo* adherence, as opposed to mechanical trapping, of lymphocytes entering lymph nodes. To detect GFP parasites, images at five different depths per stack were acquired during 1.5 min with a velocity of 0.3 s per stack in spleens from mice with 17X and 17XL infections at 1% and 10% parasitaemia representing, respectively, days 3 and 4 p.i. (Movies S2 and S3). Moreover, control animals injected with FITC-labelled RBCs were used to characterize the movement of these cells in normal spleens. Different parameters to describe particle movement were quantified by 3D manual tracking with the help of Z-coded colour stacks to provide depth information (Fig. 3D–F and Movie S4). Quantitative analysis was performed using images from mice infected with both lines of parasites on day 3 p.i, corresponding to 1% parasitaemia. Under these conditions, individual particles are easily followed. Uninfected mice were injected with the necessary amount of labelled RBCs to achieve the same percentage of fluorescent cells. Noticeably, the mean velocity of 17X parasites was significantly lower than that of 17XL parasites (*P* < 0.001) (Fig. 3G). Moreover, pRBCs in the vessels had the same velocity as surrounding RBCs visualized by reflection (Fig. 3C), suggesting differences in velocity of movement outside the vessels. In addition, 17X parasites showed a loss of directionality with respect to 17XL parasites, as evidenced by the distribution of particle population by distance versus path length (*P* < 0.001) (Fig. 3H). The lack of directionality was accompanied by an increase in the residence time of parasites in a given area of the spleen. Indeed, the median time in which a parasite appeared was 6.83 times higher for non-lethal parasites than for lethal parasites (*P* < 0.001) (Fig. 3I). Of note, the number of parasites from the non-lethal strain that remained in the spleen for more than 20 s was 6.4 times higher than that of lethal parasites. When we analysed the same parameters for FITC-labelled RBCs, we found no significant differences to those obtained for particles from the 17XL *P. yoelii* parasites (*P* > 0.05) (Fig. S4). Of importance, *in vivo* images of the passage of the non-lethal strain showed an adhesive, rolling-circle behaviour of pRBCs in real time, further suggesting that these changes in motility were due to adherence and not to retention by spleen cells (Movies S5 and S6).

### *In vitro* adherence of pRBCs to spleen-derived fibrocytic cells and transmission electron microscopy further indicate adherence to the spleen

**Fig. 4:**
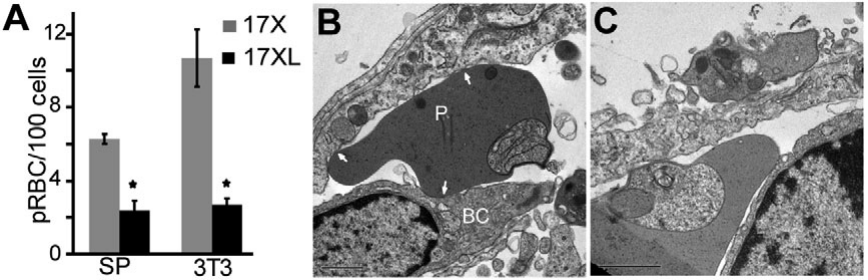
Adhesion of *P. yoelii* 17X pRBCs to fibroblastic cells. A. *In vitro* adhesion assays of 17X and 17XL pRBCs on cultured fibrocytic splenocytes (SP) and 3T3/NIH fibroblasts. Experiments were performed in duplicate and data expressed as the mean number of pRBC ± SEM per 100 cells. Differences are statistically significant (*P < 0.05). B, C. Ultramicrographs of the spleen of mice infected with the non-lethal line at 1% parasitaemia. pRBCs (P) were found in close contact with elongated fibroblastic structures corresponding to barrier cells (BCs). Adhesion of pRBCs to tissue BCs was evidenced by points of P membrane deformability and close interaction with BC membrane, indicated by arrows. Scale bars represent 1 µm.

To seek further evidence of adherence of pRBCs infected with 17X parasites in the spleen of Balb/c mice, we isolated splenocytes and cultured them in conditions where adherent cells were mostly fibrocytic splenocytes (Borrello and Phipps, 1996). Significantly, pRBCs infected with 17X on day 4 p.i. showed a 2.7-fold higher adhesion rate to fibrocytic splenocytes than those infected with 17XL (*P* < 0.05). Similar results (fourfold increase, *P* < 0.05) were obtained from adhesion assays using the immortalized cell line of fibroblastic origin 3T3/NIH, showing further evidence of the adherence capacity of RBCs infected with the *P. yoelii* 17X strain (Fig. 4A). Moreover, transmission electron microscope (TEM) images of spleens after infection with 17X showed pRBCs in close contact with longitudinal structures of fibroblastic cells that shared morphological features with those described in spleen barrier cells (Weiss *et al*., 1986) (Fig. 4B and C). This phenomenon was not observed in TEM analysis of spleens from animals infected with 17XL. Together, intravital imaging, *in vitro* adherence to fibrocytic cells and TEM results strongly suggest adherence of pRBCs to the spleen in this rodent malaria model.

### Expression of fibroblast growth factor 8 (FGF8) associates with the formation of a spleen tissue barrier in infections with 17X parasites

**Fig. 5:**
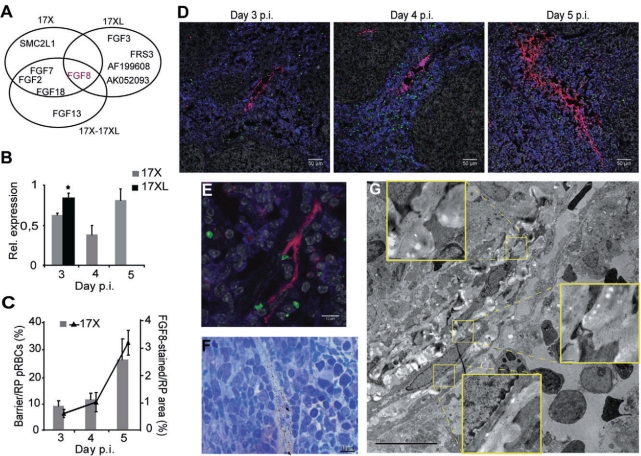
Identification of FGF8 as barrier cell marker and histopathological analysis of infected spleens. A. Venn diagram of the relations between the three sets of the five most variable genes in lines 17X (Cy3), 17XL (Cy5), and difference between lines (Cy3–Cy5), obtained from the microarray expression analysis of the spleen of Balb/c mice infected with 17X and 17XL at 3–5 days p.i. B. Real-time PCR analysis of FGF8 expression in the spleen of mice infected with the two strains. Values are expressed as the mean ± SEM of two series of samples normalized to β-actin and expressed in relative amounts with respect to amplification in samples from uninfected spleens. Differences between the two lines are statistically significant *P < 0.05. C. (Right axis, lines) Quantification of FGF8-stained area on immunostained spleen cryosections, normalized to the red pulp area of the tissue section measured and expressed in percentage. (Left axis, bars) Quantification of the numbers of parasites within the FGF8-positive stained area expressed as a percentage of the number of parasites within the red pulp. Data correspond to the mean ± SEM of three images of the spleens from two series of mice infected with 17X line on days 3, 4 and 5 p.i. D. Time-series immunohistofluorescence of spleens of mice infected with 17X parasites on days 3 to 5 p.i. Staining for FGF8 (red), F4/80 macrophages (blue), GFP (green) and DNA (gray) is shown. Scale bars represent 50 µm. E, F, G. Microscopic visualization of FGF8 expression in spleens infected with the non-lethal strain of *P. yoelii*. E. Immunohistofluorescence of spleen infected with 17X at day 3 p.i. visualized by confocal microscopy at 63× magnification. F. Silver enhanced image of a FGF8 immunogold stained semithin section showing FGF8 (arrows). G. Immunoelectron microscopic analysis of FGF8 expression in the spleen of mice infected with 17X at 1% parasitaemia with blow-ups of selected areas in yellow boxes (scale bar 3.5 µm). Scale bar represents 10 µm (E, F, G).

The blood spleen barrier is formed by cells of fibroblastic origin (Weiss *et al*., 1986). We thus performed time-series global transcriptional analyses from spleens of mice infected with 17X and 17XL parasites on days 3, 4 and 5 p.i using commercially available arrays representing the complete mouse genome (Agilent Whole Mouse Genome G4122A). Initially, low-variance filtering of genes across each strain was performed and some relevant genes were identified and grouped into functional families, among which erythropoiesis, glycolysis and fibroblasts can be highlighted (all data deposited at GEO; accession number, GSE17603). Then, the five most variable fibroblast genes (from a total of 67 in this array) were extracted for each strain and between them. The most salient feature of gene expression values was that 17X had the largest variations throughout this time-course experiment (Fig. S5). Significantly, the gene encoding the mouse FGF8 was found to be the only gene in all three sets of genes with the largest variability (Fig. 5A). Accordingly, we decided to further analyse this particular gene and logFC values were validated by real-time polymerase chain reaction (PCR) analysis (Fig. 5B).

To determine whether FGF8 might be a molecular marker to the spleen tissue barrier, we used an anti-FGF8 murine polyclonal antibody for immunohistofluorescence analysis. Quadruple labelling of GFP-expressing parasites, FGF8, F4/80 red pulp macrophage receptor and nucleic acids was performed on time-series cryosections of the spleens of uninfected and mice infected with the non-lethal and lethal lines. Visualization under laser confocal microscopy revealed the existence of distinct FGF8-stained longitudinal structures only in the spleens of animals infected with 17X (Figs 5D and S6). Quantitative data in stained areas reflected increased expression of FGF8 over days 3, 4 and 5 p.i., which correlated with the percentage of non-lethal parasites found in association with FGF8-stained structures in the red pulp (Fig. 5C and E). Remarkably, 25% of the non-lethal parasites are associated to the FGF8-stained structure representing *c*. 3% of red pulp at day 5 p.i. In contrast, spleens of mice infected with 17XL showed the presence of small structures that did not develop into barriers. No specific staining was detected in liver or kidney sections from the same animals (Fig. S6), further demonstrating that expression of FGF8 is only associated with formation of this organ-specific tissue barrier. Moreover, studies using correlative microscopy to localize FGF8 at the ultrastructural level showed specific staining in the intercellular spaces surrounding barrier cells of spleen micrographs (Fig. 5F and G).

### MRI confirms remodelling of the spleen in the 17X strain

**Fig. 6:**
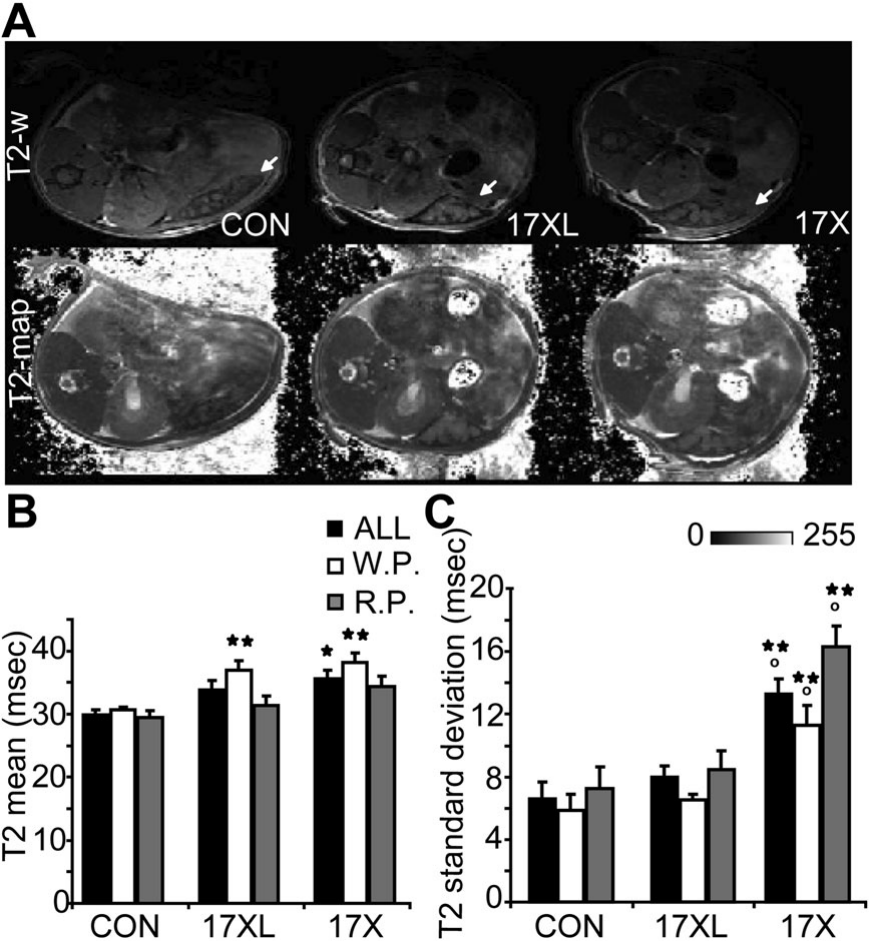
MRI of the spleen using T2 relaxometry. A. Coronal T2-weighted images (top) and T2 maps (bottom) showing the spleen (arrows), right kidney and back muscle from an uninfected mouse (CON), a mouse infected with the lethal line and a mouse infected with the non-lethal line on day 4 p.i. Gray-scale bar in T2 map represents intensity-related T2 values from 0 to 255 msec. B. Measures for mean T2 relaxation time were evaluated in the white pulp (W.P.), red pulp (R.P.) or total area of the spleen, as well as in the muscle and kidney (Fig. S8). C. Variance of T2 values in the selected areas. T2 times are expressed in milliseconds (msec) as the mean ± SEM of 4 to 5 mice from each group; data were evaluated by analysis of variance versus uninfected (*P < 0.05 and **P < 0.01) and versus lethal (*P* < 0.01) (Tukey *post hoc* test).

Magnetic resonance imaging is a non-invasive technique that allows the study of the structural and functional features of tissues and organs. To determine whether or not MRI might also be of value in detecting structural differences in the spleens of mice infected with lethal and non-lethal lines of parasites, we measured spleen T2 relaxation times, which can provide accurate quantitative measurements to differentiate between normal and pathological tissues as well as information on cellularity and oedema (Oh *et al*., 2006). Noticeably, MRI of Balb/c spleens demonstrated a significant increase in T2 relaxation times in mice infected with the 17X strain compared with uninfected mice (*P* < 0.05) (Fig. 6A and B). In addition, the standard deviation of T2 relaxation times measured in the red and white pulp of mice infected with the 17X strain was almost twice as high as that of control mice and mice infected with the 17XL strain (*P* < 0.01), further indicating enhanced structural heterogeneity after infection with the non-lethal strain (Fig. 6C). None of these differences were observed in similar measurements of selected areas of muscle or kidney from the same animals (Fig. S7). These findings corroborate that only the *P. yoelii* 17X strain induces spleen remodelling.

## Discussion

**Fig. 7:**
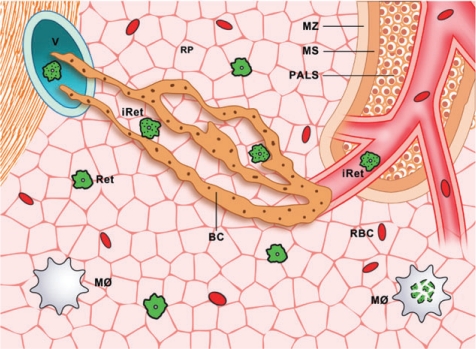
Model of spleen-clearance evasion mechanism in reticulocyte-prone non-lethal malaria. Infection of Balb/c mice with the reticulocyte-prone non-lethal *P. yoelii* 17X strain induces remodelling of the spleen through the formation of a spleen tissue barrier of fibroblastic origin characterized by a syncitium of cells. Such barrier facilitates the channelling of blood from arterioles to venules and the adherence of infected reticulocytes to it thus physically protecting them from destruction by macrophages. V, venous lumen; Ret, reticulocytes; iRet, infected reticulocytes; RP, red pulp; PALS, periarteriolar lymphoid tissue; MS, marginal sinus; MZ, marginal zone; MØ, macrophages; BC, barrier cells.

The spleen is exquisitely adapted to selectively clearing abnormal RBCs, particles from the blood and infectious agents including malaria. Because of ethical and technical constraints, however, functional studies of the spleen are very limited and none to date has addressed the dynamic features of the passage of the malaria parasite through this organ *in vivo*. In here, we used the Balb/c *P. yoelii* rodent malaria model to study the function and structure of the spleen using different techniques including intravital and MRI. Our findings support a model in which the reticulocyte-prone non-lethal *P. yoelii* 17X strain induces remodelling of the Balb/c spleen through the formation of a spleen blood barrier of fibroblastic origin where infected RBCs adhere and where macrophage-clearance escape is facilitated (Fig. 7, Movie S7).

The different phenotypes of the *P. yoelii* reticulocyte-prone non-lethal versus normocyte-prone lethal lines are characterized by numerous differences including sub-cellular localization of parasite proteins, invasion mechanisms, parasite growth rates and induction/suppression of immune responses (Pattaradilokrat *et al*., 2008; 2009; Otsuki *et al*., 2009). In our studies, we decided to examine the phagocytic activity of macrophages within the red pulp as this is where infected RBCs are destroyed (Yadava *et al*., 1996) and also because other studies have shown the importance of spleen macrophages in parasite control during the early phase of infection in both lethal and non-lethal strains of *P. yoelii* (Couper *et al*., 2007). In addition, several *in vitro* studies evaluating macrophage activity have shown that macrophages from animals infected with different non-lethal malaria parasites have a greater capacity to generate O_2_ metabolites and greater cytotoxic activity than macrophages obtained from mice infected with lethal species (Brinkmann *et al*., 1984; Taverne *et al*., 1986). We observed lower numbers of parasites within macrophages in infections with 17X than in infections with 17XL. However, because these numbers were observed together with larger numbers of parasites in spleens infected with 17X and no differences were observed in either *in vitro* or *in vivo* phagocytic activity measured using fluorescent beads between the two infections, it can be assumed that the differences in the numbers of parasites within the macrophages are not due to an increased ability of macrophages to kill parasites during non-lethal infections. This provides yet further evidence of different spleen compartmentalization in animals infected with non-lethal strains.

Cytoadherence of infected RBCs to the endothelium of venular capillaries in the deep vascular bed of inner organs is considered a fundamental aspect in the pathology of malaria and a major mechanism to escape spleen clearance (Miller *et al*., 1994). Remarkably, in our study 27% of the parasites from the non-lethal line remained in the spleen for more than 20 s, lost directionality, reduced their velocity and showed rolling-circle behaviour, all parameters associated with adherence (Grayson *et al*., 2003; Khandoga *et al*., 2009). Adherence of 17X parasites was also demonstrated *in vitro* using spleen-derived fibrocytic cells and immortalized fibroblast cells, as well as by TEM analysis demonstrating physical contact of pRBCs to barrier cells. Noticeably, in all these experiments, 17XL parasites served as a stringent control for specificity of such adherence. Thus, even though we have not formally proven the existence of ligand-receptors in this adherence, mechanical retention is difficult to accept as the sole explanation of our results, particularly if we consider that the spleen of mice is non-sinusoidal and the pore size of the reticular mesh in the red pulp is several times that of the diameter of pRBCs (Groom *et al*., 2002). We thus believe that our findings provide, to our knowledge, the first strong evidence of adherence of malaria parasites to the spleen opening new avenues for the investigation of host–parasite interactions in this lymphoid organ.

A question that remains to be answered is what role, if any, FGF8 plays in the formation of the spleen barrier. Fibroblast growth factors are peptides involved in signalling for correct vertebrate development *in vivo* and determining their function has presented major difficulties as many knock-out studies are lethal in embryonic stages or do not cause any particular phenotype as a result of the redundancy of this family (Itoh, 2007). It is tempting to speculate that the initial adherence of infected RBCs to individual spleen barrier cells transduces a signalling pathway via its specific FGF nuclear receptors to express FGF8, which, in turn, would induce autocrine activation and proliferation to create the barrier. To explore this possibility, we treated mice with PD 173074, an inhibitor of the FGFR3 receptor, known to be activated by FGF8 (Eswarakumar *et al*., 2005), using a regimen of 25 mg kg^−1^ of PD29447 (Sigma) from day −1 to day 14 p.i. Immunostaining for FGF8 of treated mouse spleens revealed that the tissue barrier developed in the non-lethal line, suggesting that a more complex mechanism is involved (not shown). Further studies are required to establish the role of FGF8 and other factors in the formation of this blood spleen barrier.

The spleen appears to have a dual role in malaria infections as it destroys pRBCs but also appears to modulate parasite biology as splenectomy has a major effect on the parasite phenotype (Engwerda *et al*., 2005). Strikingly, retention of *Plasmodium falciparum* RBCs containing ring stages by the human spleen was recently demonstrated in an *ex vivo* model system (Safeukui *et al*., 2008). Moreover, the same authors postulated that retention of rings in the spleen reduces the risk of cerebral malaria while increasing the risk of severe malarial anaemia (Buffet *et al*., 2009). The results presented here reveal remodelling of the spleen and adherence to this organ in Balb/c mice infected with the *P. yoelii* 17X strain. Moreover, they suggest that structural remodelling of the spleen might have a role in chronic infection as it is somehow related to delay in the onset of precrisis and prevention of host death. Noticeably, injection of splenocytes from *P. yoelii* 17X-infected mice that had cleared parasitaemia for over 3 months into naïve recipient mice resulted in infection in one of three mice (data not shown). While further experimentation is required to support this single observation, it indicates that in this rodent model, *P. yoelii* can establish sub-patent chronic infections as has been previously observed in CBA mice (Jayawardena *et al*., 1975). It will be interesting to determine the intercellular communication signals that induce this remodelling process and the ligand-receptors involved in adherence. Importantly, remodelling was also evidenced using MRI, a non-invasive technique widely used in human diagnostics. Finally, we postulate that a similar mechanism occurs in *Plasmodium vivax*, a reticulocyte-prone non-lethal human malaria parasite, as cytoadhesion has been recently reported to occur in cells expressing endothelial receptors (Carvalho *et al*., 2010).

## Experimental procedures

### Mice and parasites

All the animal studies were performed at the animal facilities of Hospital Clinic in Barcelona in accordance with guidelines and protocols approved by the Ethics Committee for Animal Experimentation of the University of Barcelona CEEA-UB (Protocol No DMAH: 3968). Female Balb/c mice, 6–8 weeks of age, were obtained from Charles River Laboratories. The original *P. yoelii yoelii* lethal (17XL) and non-lethal (17X) strains were obtained from MR4 (http://www.mr4.org/). *P. yoelii*–GFP transgenic lines of 17XL and 17X were generated using the same vectors, targeting strategy and protocols described elsewhere for *P. berghei* (Franke-Fayard *et al*., 2004). Infections were induced by the i.p. injection of 5 × 10^5^ pRBCs obtained from the tail blood of donor mice at 5–10% parasitaemia. Parasitaemia was monitored daily by Giemsa staining of blood smears calculating the percentage of pRBCs over total RBCs in three optical fields of approximately 300 RBCs. Brilliant Cresyl Blue-Giemsa staining was used to detect reticulocytes (Taylor-Robinson and Phillips, 1994). Briefly, 5 µl of mouse blood was mixed with an equal volume of 1% Brilliant Cresyl Blue in 0.65% sodium chloride and incubated at room temperature (RT) for 20 min. After incubation, smears were prepared and left to air dry. After fixation with methanol, the smears were counterstained with Giemsa. Infected and uninfected reticulocytes were counted in different optical fields with, at least, 1500 RBCs.

### Tissue preparation

Spleens, livers and kidneys from infected (17XL and 17X) and uninfected mice were aseptically removed on days 1 to 5 p.i. Tissues were fixed in 4% paraformaldehyde at 4°C for 2 h and cryoprotected in 30% sucrose at 4°C overnight before inclusion in Optimum Cutting Temperature compound (O.C.T. Tissue-Tek) and storage at −80°C. Cryosections of 7 µm were cut and processed for histopathological analysis.

### Immunofluorescence assays

Tissue sections were thawed at RT for 30 min and fixed in cold acetone for 5 min. Fixed sections were treated with 100 mM NH_4_Cl PBS for 5 min and 100 mM Glycine PBS for 10 min to reduce autofluorescence. After blocking with 1% BSA PBS at RT for 1 h, sections were incubated at 4°C overnight with primary antibodies diluted in 0.5% BSA and 0.05% Tween-20 PBS. The primary antibodies used were rabbit anti-GFP antibody (Invitrogen, dilution 1/100), rat anti-F4/80 antibody (Abcam, dilution 1/100) and goat anti-FGF8b antibody (R&D Systems, dilution 1/7). After incubation, tissue sections were washed in 0.05% Tween-20 PBS and incubated with secondary antibodies at RT for 1.5 h. The corresponding secondary antibodies were conjugated to Alexa Fluor 488, 647 and 546 (Invitrogen, dilution 1/200). After washing, nuclei were stained with 4,6-diamidino-2-phenylindole (Invitrogen, 5 mg ml^−1^) at RT for 7 min. Sections were mounted in Vectashield mounting media.

Images were obtained using a Leica TCS-SP5 microscope at a magnification of 20x (0.7 NA) and 63x (1.4 NA, oil objective). Five Z-stacks covering a depth of 7 µm were acquired and images were processed using ImageJ software (version 1.39o, Wayne Rasband, NIH, http://www.macbiophotonics.ca). Z-maximum projection and filtering of Gaussian Blur = 1 was performed for all images. For each day p.i., the FGF8-stained area over the red pulp area was quantified using ImageJ software. A threshold was set for positive staining and values averaged over three images obtained at 20× magnification representing different areas of the immunostained section.

### Tissue Giemsa staining

Fixed spleen cryosections were stained in 20% Giemsa solution, 0.1% acetic acid and 96% ethanol and finally dehydrated and mounted in Depex-Polystyrene dissolved in xylene mountant for microscopy (Sigma-Aldrich). To determine the red pulp to white pulp ratio, those two regions were manually defined on images representing the whole Giemsa-stained tissue sections, considering the differential histopathological and Giemsa staining patterns between the red and white pulp. The area of the regions was then calculated using ImageJ software.

### Quantification of number of parasites in spleens

Fluorescence wide-field microscopy was used to acquire images of the spleen and liver sections immunostained with anti-GFP antibody. Giemsa-stained sections were visualized under polarized light microscopy at 20× magnification. For parasite quantification, images were thresholded and automatic particle counting was used in ImageJ software, with a particle size of > 2 µm and circularity values of 0–1. The number of particles in the red pulp of the spleen was normalized for the manually defined tissue area. To determine the number of GFP parasites that were associated with the tissue barrier, Z-projections of the confocal images were used to define a second region of interest based on the FGF8-stained area within the red pulp. This selection was dilated two times to ensure inclusion of all associated pRBCs and number of parasites calculated as above.

### Flow cytometry analysis of macrophage activity

Splenocytes were prepared from the spleens of mice infected with 17X and 17XL GFP transgenic parasites on day 4 p.i. Briefly, the spleens were homogenized and passed through a nylon mesh to create a single-cell suspension. Before addition of specific fluorescently labelled antibodies, the cells were preincubated with Seroblock anti-Fc receptor antibody (Abcam, dilution 1/100) for 10 min. Splenocytes were incubated with an antibody against murine F4/80 receptor conjugated to Alexa Fluor 647 (Invitrogen, dilution 1/10) at 4°C for 30 min. Cells were washed twice with 5 mM EDTA, 1% BSA PBS. Samples were incubated with the viability dye propidium iodide (Sigma, 1 mg ml^−1^) before data acquisition. The data were collected and analysed using a FacsCANTO flow cytometer (BD Biosciences) and CELLQuest software. GFP^+^ cells in these spleens were gated with respect to splenocytes obtained from infections with non-transgenic lines of *P. yoelii*. Data for F4/80^-^/GFP^+^, parasites and F4/80 macrophages in the spleen are representative of 200 000 events collected. For F4/80^+^/GFP^+^cell analysis, 1000 events of that population were collected.

Analysis of the phagocytic capacity of F4/80^+^ cells using fluorescent beads was performed using Balb/c mice infected with wild-type 17XL and 17X strains. *In vitro* phagocytic activity was evaluated as the capacity of macrophages to incorporate 1 µm yellow-green fluorescent beads (Molecular Probes). Splenocytes of mice infected with the two lines of parasites were resuspended to 1 × 10^8^ cells ml^−1^ in DMEM containing 5% heat-inactivated FBS and seeded on a 24-well plate. Plates were incubated at 37°C, 5% CO_2_, 100% humidity for 1 h. They were then incubated with 100 beads per cell in 500 ml medium at 37°C for 1 h. Cells were then washed three times with cold PBS to remove any free beads and scraped from the plate. Cells samples were labelled with an anti-F4/80 antibody conjugated to Alexa Fluor 647 and analysed on a FacsCANTO flow cytometer. The acquisition threshold was set to include cells but to exclude debris and remaining unbound beads. For *in vivo* analysis of F4/80^+^ macrophage activity, mice were anaesthetized before intravenous (i.v.) injection of 200 µl of PBS with 1.7 × 10^8^ 3 µm yellow-green fluorescent beads (Polysciences). After 30 min, the mice were killed by cervical dislocation and the spleen was removed and processed as described above. Splenocytes were incubated with an anti-F4/80 antibody conjugated to Alexa Fluor 647 and 10^6^ cells per sample were analysed on a FacsCANTO flow cytometer. The percentage of phagocytic cells (macrophages that had ingested beads) was calculated as a function of F4/80^+^ cells.

### Parasite *in vivo* imaging and mobility analysis

*In vivo* imaging of the spleen was performed on mice infected with the 17XL or 17X GFP transgenic lines at 1% (day 3 p.i.) and 10% (day 4 p.i.) parasitaemia. Briefly, the spleen of anaesthetized mice (100 mg kg^−1^ ketamine and 5 mg kg^−1^ midazolam i.p.) was surgically exposed as described elsewhere (Thiberge *et al*., 2007). Imaging of living parasites within the intact spleen was achieved by placing the mice on a stage of a Leica TCS-SP5 microscope fitted with an inverted 63x (1.3 NA) glycerol objective, which permits a working distance of 0.28 mm. Movies of 1.5 min were recorded through five Z-stacks covering a depth of 8 µm at a speed of 8 kHz. Fluorescence was recorded on two different channels (excitation/emission wavelength 488/505–580 nm for GFP and of 570–630 nm for tissue autofluorescence) with the pinhole set to 3.0 Airy units. RBC reflection (488/480–495 nm) was used in some experiments to obtain additional information on the zone being imaged and in the blood flow experiments described below.

Quantization of parasite mobility parameters was achieved using movies of the spleen of infected mice at 1% parasitaemia and of FITC-RBC injected control mice using ImageJ software. To this end, 4D (x, y, z, t) manual tracking of green fluorescent particles was performed using the MTrackJ plugin (written by E. Meijering) with the help of Z-coded colour stacks to facilitate single-particle identification and tracking over time. Particles representing pRBCs from each strain were tracked in different areas of the spleen of mice at 1% parasitaemia. In total, 120 particles from three mice infected with each parasite line were tracked. Tracking was performed in a maximum of 100 frames for each particle. Z movement was considered only if displacement was higher than 6 µm (average diameter for an infected particle). For each particle, measures for directionality, defined as the quotient of displacement versus path length (Miller *et al*., 2002), mean velocity and residence time were calculated and plotted as density distributions. Differences between the two lines were assessed using the equality-of-medians test in stata (IC10).

### Spleen blood flow measurements

After exposing spleens as described above, 50 µg of 70 kDa Dextran labelled with Texas Red (Invitrogen) dialysed through a 0.2 µm membrane and diluted in 100 µl of saline buffer were injected i.v. Image was further acquired using xy and xt line-scanning modes in the central lumen of the vessel as previously described (Molitoris and Sandoval, 2005; Zhong *et al*., 2008). Vessels were set horizontally, in the direction of laser scanning, by optical field rotation (not affecting speed). Bidirectional scanning with a line average of 32 was used at a speed of 8 kHz and an image of 512 pixels × 512 lines was obtained. In these images, the streaks resulting from moving cells were used to quantify blood flow as described (Zhong *et al*., 2008).

### Microarray global expression analysis

Spleens of mice infected with 17XL and 17X parasites were aseptically removed and snap-frozen in liquid nitrogen between days 3 and 5 p.i. Tissues were homogenized individually and RNA was extracted using the TrizolR reagent (Invitrogen). Total RNA was labelled using an Agilent Low RNA Input Fluorescent Linear Amplification Kit. mRNA from the spleen of each mouse infected with either the 17X or 17XL lines on days 3, 4, and 5 p.i. was labelled with Cy3 and Cy5 respectively. Dual hybridizations were performed using the Agilent Whole Mouse Genome G4122A microarray according to the manufacturer's protocol. Microarray images were obtained using the GenePix 4000B scanner. Data were processed with the Bioconductor limma package, using background correction (‘normexp’) and ‘quantile’ normalization between arrays for final values (Cy3 and Cy5 signal intensities were separately normalized as one-colour arrays).

### Real-time PCR analysis

Total RNA from spleen cells was retro-transcribed and cDNA was subjected to real-time PCR using specific primers for FGF8 and β-actin with the use of ABI-Prism 7500 (AME Bioscience). PCR was performed using the following cycling parameters: activation at 95°C for 10 min; PCR cycling, 40 cycles at 95°C for 15 s, and 60°C for 1 min. The primers used were the following: TTCCTCAACTACCCGCCCTTCA and GCCCCTCCCCTTTGCTGTGC for FGF8 and GCGGGCGACGATGCT and AGGGCGGCCCACGAT for β-actin, which hybridize to the corresponding murine cDNAs. Experiments were performed in triplicate. The PCR arbitrary units of each gene were defined as the mRNA levels normalized to the β-actin expression level in each sample.

### Electron microscopy

For ultrastructural analysis, the spleens of uninfected mice and mice infected with 17XL and 17X strains at 1% parasitaemia were aseptically removed and immediately fixed with Karnovsky's fixative. Small pieces of 1 mm^2^ were fixed in the same fixative at 4°C for at least 24 h, post-fixed in 1% osmium tetroxide and dehydrated in acetones before embedding in Spurr resin. Semithin (0.5 µm) and ultrathin (70–90 nm) sections of the spleen were obtained on an ultramicrotom Ultracut E (Reichert-Jung) equipped with a diamond knife (Diatome). Semithin sections were stained with methylene blue and optical microscopy was used to locate red pulp area. Serial ultrathin sections from the same region were cut and stained with 2% uranyl acetate and lead citrate (Reynolds, 1963) to allow high-contrast imaging. Image acquisition was performed using a JEOL 1010 TEM operating at 80 kV with a Bioscan 792 camera (Gatan MultiScan cameras).

### Correlative light electron microscopy

Correlative light electron microscopy was used to detect FGF8 expression at the ultrastructural level. Briefly, the spleens of infected mice were fixed in 4% paraformaldehyde and 0.1% glutaraldehyde phosphate buffer. Pieces of 0.5 cm were cut, dehydrated in ethanols by progressive lowering of the temperature to −35°C and embedded in Lowicryl K4M (Carlemalm *et al*., 1982; 1985;). Immunogold assays were performed on semithin sections from different blocks of the spleen covered with a blocking buffer of 1% BSA (Sigma), 20 mM glycine in 0.1M PBS, pH 7.4 at RT for 30 min in a moist chamber. After blotting off excess buffer, sections were incubated in primary anti-FGF-8b antibody diluted 1/3 with blocking buffer at RT for 2 h. Sections were washed and incubated in 10 nm protein A gold conjugate (Utrecht University) for 1 h. After washing with PBS and deionized water, the sections were silver-enhanced using a SEKL 15 silver enhancing kit (BBInternational) according to the manufacturer's instructions, with progress being periodically monitored using a light microscope. Silver enhancement was reached at between 8 and 10 min. The sections were then washed in several changes of distilled water. Next, serial ultrathin sections obtained from the blocks that revealed FGF8-specific staining were immunolabelled and prepared for TEM as above.

### *In vitro* adhesion assays

Spleen-derived fibroblasts were obtained from long-term splenocyte culture with slight modifications (Borrello and Phipps, 1996). Briefly, a single-cell suspension of the spleen from a control Balb/c mouse was obtained and cells cultured in RPMI complete medium [RPMI1640 with L-glutamine (Sigma) containing 10% fetal bovine serum (Invitrogen), 5 × 10^−5^ M 2-ME (Invitrogen), 10 mM Hepes (Sigma), 50 µg ml^−1^ gentamicin (Invitrogen), 1% Penicillin/Streptomycin (Invitrogen) and 25 mM sodium bicarbonate (Sigma)] using an incubator with 5% CO_2_ at 37°C. After 2 weeks, adherent cells were dissociated with a 0.05% trypsin-EDTA solution, seeded in 8-well chambered slides (Lab-Tek II) at 10^5^ cells well^−1^ in 300 µl well^−1^ of RPMI complete medium and incubated in 5% CO_2_ at 37°C for 2 days before adhesion assays were performed.

Murine fibroblasts of the 3T3/NIH cell line were originally obtained from ATCC (http://www.atcc.org) and maintained in culture following standard methodologies. For adhesion assays, 10^4^ cells well^−1^ were seeded in 8-well chambered slides and incubated for 2 days in 5% CO_2_ at 37°C.

For parasite preparation, total peripheral blood was collected in EDTA/PBS through intracardiac puncture from 17X- or 17XL-infected mice on day 4 p.i. The blood pellet was washed three times in RPMI adhesion media (RPMI 1640 with L-Glutamine containing 20 mM Hepes and 50 µg ml^−1^ Gentamicin at pH 6.8) and pRBCs were enriched for mature stages using a 55% Histodenz (Sigma)/PBS gradient as described (Mota *et al*., 2001). The adhesion assays were performed in duplicates, incubating the cells with 10^5^ pRBC well^−1^ in 300 µl well^−1^ of adhesion media at 37°C for 1 h. Slides were washed by immersion 10 times in adhesion media, air-dried, fixed with methanol and stained with Giemsa. The number of pRBCs was averaged from 150 optical fields of each well using an optical microscope with a 100x objective.

### *In vivo* MRI

A high-field magnetic resonance system was used for non-invasive imaging of the spleen of anaesthetized mice infected with either the 17XL or 17X line on day 4 p.i. MRI scans were performed under isoflurane anaesthesia using a BioSpec 70/30 horizontal animal scanner (Bruker BioSpin) equipped with a 12 cm inner diameter actively shielded gradient system (400 mT m^−1^). Coil configuration consisted of a surface transceiver coil for cardiac and abdominal imaging. The animals were placed in a prone position in the holder with a nose cone for administering anaesthetic gases and fixed using adhesive tape. Tripilot scans were used for accurate positioning of the animals' spleen inside the isocentre of the magnet. As a reference to select the same slices in the T2 transversal relaxation maps, T1-weighted 3D coronal images were acquired using a FISP (Fast Imaging Steady State Precession) sequence with the following parameters: echo times = 2.2 msec, repetition time = 14 msec, field of view = 35 × 35 × 17 mm, matrix = 256 × 256 × 50 pixels, resulting in a spatial resolution of 0.137 × 0.137 × 0.340 mm. Indeed, T2 mapping of one abdominal coronal section containing the spleen, the kidney at the level of the renal artery and the back muscle was acquired using an MSME sequence with the following parameters: 16 different echo times, repetition time = 2.5 s, field of view = 30 × 30 × 1 mm, matrix = 256 × 256 × 50 pixels resulting, in a spatial resolution of 0.117 × 0.117 × 1.00 mm.

T2 maps were constructed and analysed with Paravision 5.0 (Bruker BioSpin). Measurements of T2 relaxation times were performed by manually drawing the red and white pulp of the spleen. Three different blinded experimenters repeated this process, and an average of the three measurements was obtained. T2 values from muscle and the renal cortex were measured in independent circular regions of interest.

## Supporting information

**Fig. S1**. Characterization of the Balb/c-GFP *P. yoelii* murine malaria model (A) Transgenic *P. yoelii* parasites express GFP at all asexual blood stages (rings, trophozoites, schizonts and merozoites). (B) Time-course parasitemia in experimental infections of 6–8 week-old female Balb/c mice with the 17XL and the 17X lines. Curves were calculated from the mean of five mice injected with 5 × 10^5^ pRBCs from each line of the wild-type or GFP transgenic parasites. Time-course reticulocytemia (number of reticulocytes/number of RBCs × 100) (C) and percentage of infected reticulocytes (number of reticulocytes/number of infected RBCs × 100) (D) in GFP transgenic *P. yoelii* 17X and 17XL infections. Results are based on BCB-Giemsa staining of infected blood smears. Curves were calculated from the mean of at least six mice per group injected with 5 × 10^5^ pRBCs from each line of the GFP transgenic parasites. (E) Brilliant Cresyl Blue (BCB)-Giemsa staining images of blood smears from the transgenic 17X and 17XL infected mice on days 3, 4, 5 and 10 p.i.

**Fig. S2**. Retention of *P. yoelii* 17X and 17XL parasites in the murine spleen. (Upper panel) Visualization under polarized light microscopy of Giemsa-stained cryosections of the spleens from mice infected with the 17X line (A) and the 17XL line (B) at 1% peripheral parasitemia (day 3 p.i.). (Lower panel) Thresholded images (142–255 threshold level) used for parasite quantification, showing distribution of parasitized red blood cells in the red pulp of the spleen. N = numbers of parasites in spleen. Scale bars represent 50 µm.

**Fig. S3**. Analysis of spleen closure four days after *P. yoelii* infection. 1.68 × 10^8^ 3 µm yellow-green fluorescent beads were injected i.v. into uninfected mice (Con) and mice infected with 17X and 17XL wild-type parasites on day 4 p.i. Five minutes after injection, splenic uptake of beads was analyzed by flow cytometry (A) and fluorescence microscopy (B). (A) 10^6^ spleen cells were analyzed by FACS and the numbers of beads was quantified. The percentage of the number of beads per spleen cell is expressed as the mean ± SEM of six different mice (ANOVA). No significant differences were observed between the groups. (B) The uptake of beads per spleen area was quantified using ImageJ software in six to ten confocal images per mouse with six mice per group after injection of fluorescent beads. Each bar indicates the mean, and each error bar the SEM. No significant differences were observed between the groups after ANOVA analysis.

**Fig. S4**. Analysis of FITC-labeled RBC movement. Density distribution of FITC-labeled RBCs in the spleen of control mice (N = 3) by mean velocity (A), directionality (B) and residence time (C). In total, 100 particles were tracked using the same methodology described for GFP-pRBCs in infected mice and compared to 17X and 17XL parasites with equality-of-medians test. The median value for mean velocity is 103.66 µm/sec, for directionality 0.852 and for residence time 0.9 sec. Particles quantified were moving outside vessels (49.52%) and through vessels with a diameter of less than 10 µm (16.19%) and greater than 10 µm (32.38%).

**Fig. S5**. Microarray analysis. Heatmaps and plotted values of the logFC values on days 3, 4 and 5 p.i. (with respect to time 0) for unsupervised selection of fibroblast-related genes with the largest variability on spleen samples of mice infected with 17X(Cy3) (A); 17XL (Cy5) (B) and 17X-17XL differences (Cy3–Cy5) (C) together with a housekeeping gene (GAPDH) with small variability.

**Fig. S6**. Strain and tissue specificity of the *P. yoelii* 17X-induced barrier. Time-series immunohistofluorescence of tissue sections. (A) Sections of spleen and liver of mice infected with *P. yoelii* 17XL lethal strain. No specific staining for FGF8 was observed in cryosections of infected mice on days 3 to 5 p.i. (B) Immunofluorescence of liver and kidney cryosections in 17X infected mice was also negative for FGF8. Staining for FGF8 (red), GFP (green), F4/80 macrophage receptor in the spleen (blue) and nucleic acids (gray) was performed. Scale bars represent 50 µm.

**Fig. S7**. MRI analysis of the muscle and kidney. (A) T2 mean relaxation times quantified in circular regions of the back muscle and the outer part of kidney from T2 maps of coronal T2-weighted images containing the spleen (Figure 6). (B) Variance of T2 values in the selected areas of muscle and kidney. T2 times (msec) are expressed as the mean ± SEM of four to five mice from each group (control, lethal and non-lethal). No significant differences were observed.

**Movie S1**. IVM movie of a representative dextran-labeled (red) spleen vessel (10.4 µm lumen diameter) in a mouse infected with the 17X GFP line (green) at 1% parasitemia used for blood flow analysis. Scale bars represent 10 mm. Quick-time. S1 (4.6 MB, 9 sec).

**Movie S2**. Time-lapse intravital microscopy images of the murine spleen infected with 17XL GFP-transgenic parasites at 10% parasitemia (Z-maximum projection). Parasite and tissue autofluorescence are shown in green and red respectively. Scale bars represent 10 µm and the time interval is in sec. Quick-time. S2 (4.1 MB, 10 sec).

**Movie S3**. Time-lapse intravital microscopy images of the murine spleen infected with 17X GFP parasites at 10% parasitemia (Z-maximum projection). Parasite and tissue autofluorescence are shown in green and red respectively. Scale bars represent 10 µm and the time interval is in sec. Quick-time. S3 (2.6 MB, 10 sec).

**Movie S4**. IVM Z-coded color video of the murine spleen infected with 17XL showing three-dimensional movement of parasites. These videos were used as a tool to follow singleparticle movement over time. Each time point is the result of a projection from 5 different depths (Z) with a Z-delay of approximatively 0.06 sec. Depth code: yellow (0 µm), orange (2 µm), pink (4 µm), blue (6 µm), green (8 µm). Quick-time. S4 (0.9 MB, 3 sec).

**Movie S5**. IVM video of representative area of the spleen of mice infected with 17X at 1% peripheral parasitemia. Selected area focus on an example of the dynamic adherence of pRBCs to spleen cells. Twenty-seven percent of the parasites from the non-lethal line remained in the spleen for more than 20 sec, compared to 4% in the lethal line. Quick-time. S5 (0.7 MB, 6 sec).

**Movie S6**. IVM video of representative area of the spleen of mice infected with the 17X at 1% peripheral parasitemia. Selected area focus on examples of the dynamic adherence of pRBCs to spleen cells. Quick-time. S6 (0.6 MB, 6 sec).

**Movie S7**. Animation illustrating our present model of *P. yoelii* 17X line-induced spleen remodeling, infected RBC adherence to the tissue barrier and macrophage-clearance evasion. Quicktime. S7 (1.1 MB, 11 sec).
