# Supplementary figures and images for: Strain-specific spleen remodelling in Plasmodium yoelii infections in Balb/c mice facilitates adherence and spleen macrophage-clearance escape
Source: Cell Microbiol. 2011 Jan;13(1):109–22. doi: 10.1111/j.1462-5822.2010.01523.x (PMC3228402; doi:10.1111/j.1462-5822.2010.01523.x)

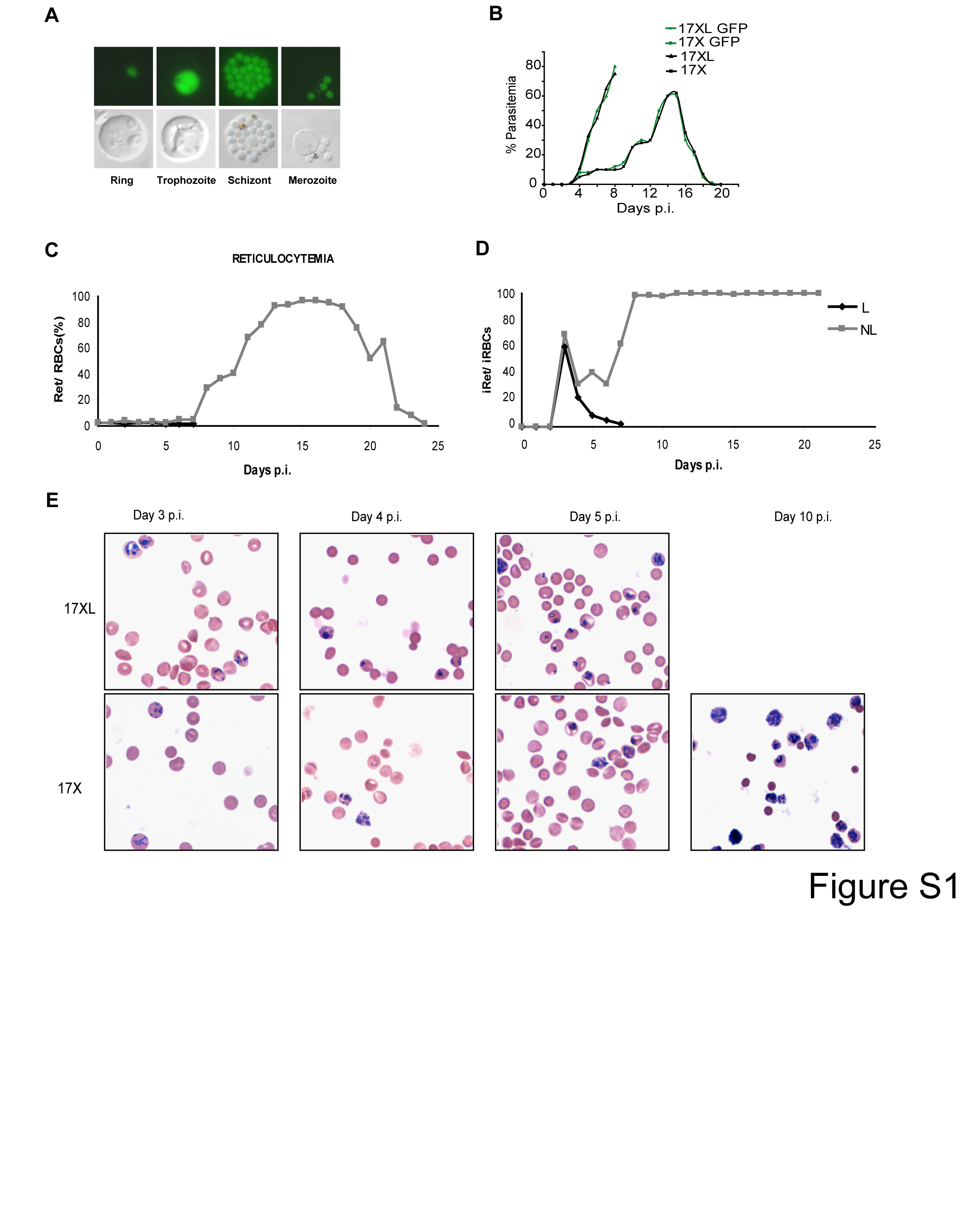

Supplement: Supplementary file 1 [file cmi0013-0109-SD1.tif]

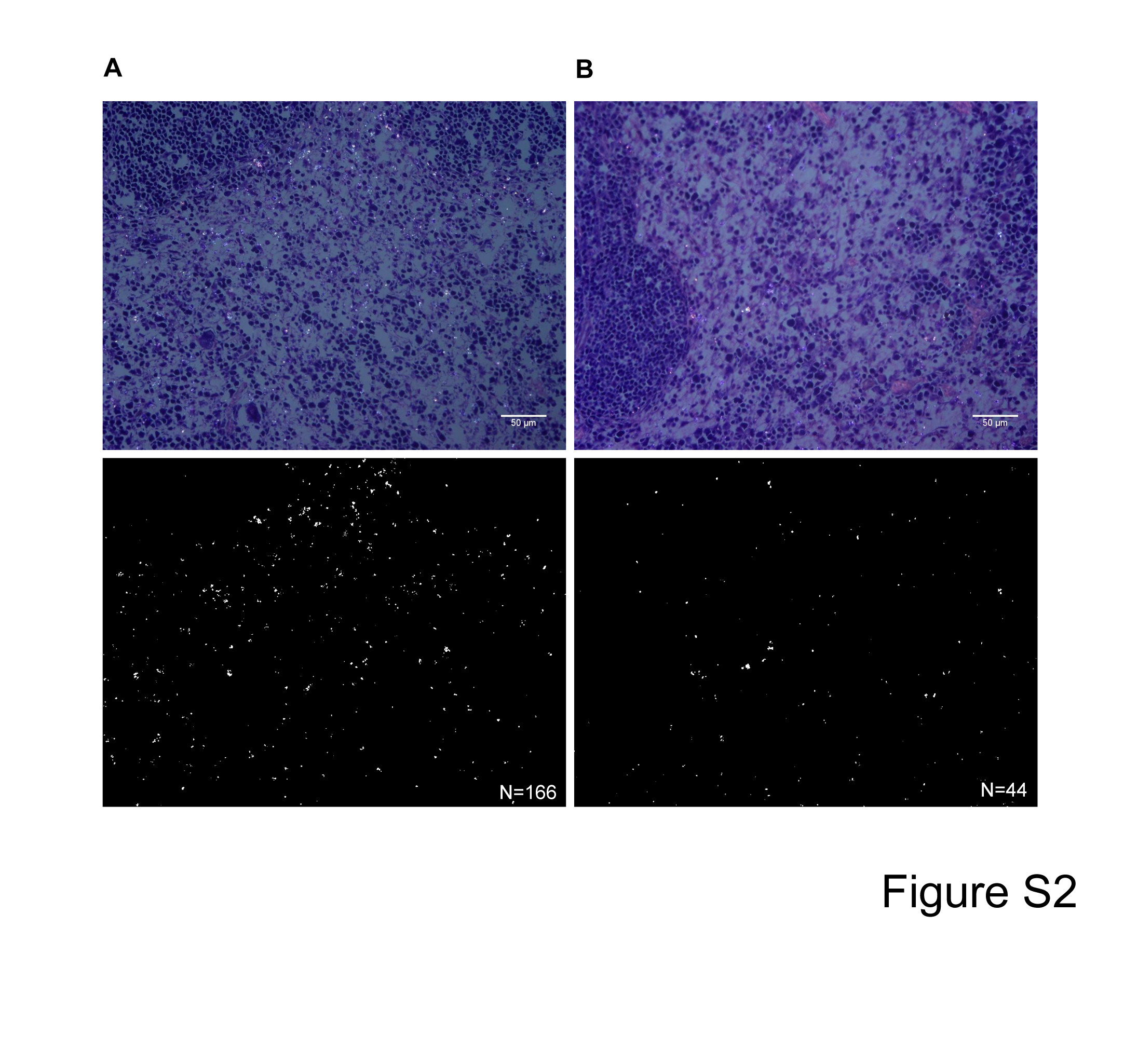

Supplement: Supplementary file 2 [file cmi0013-0109-SD2.tif]

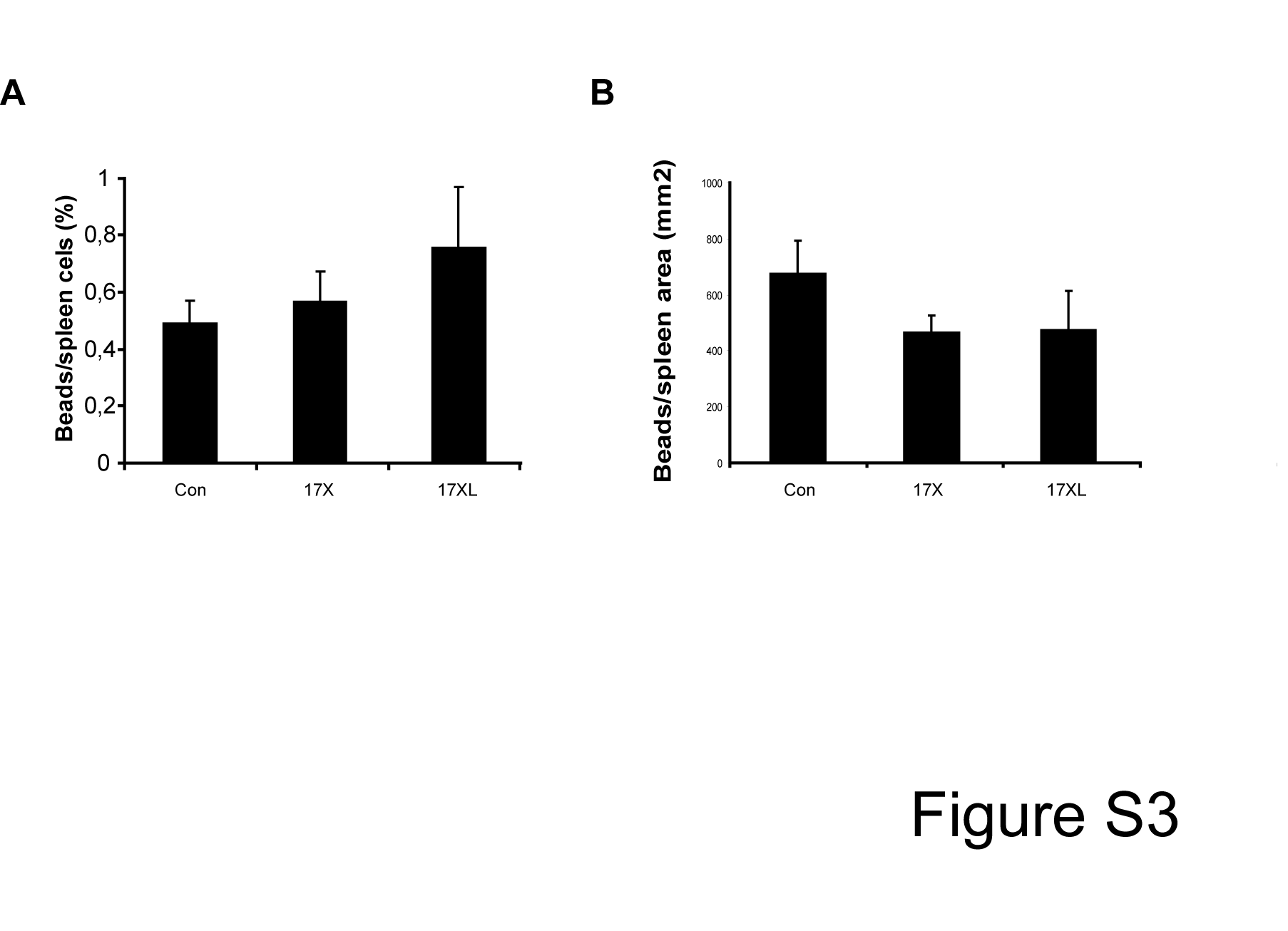

Supplement: Supplementary file 3 [file cmi0013-0109-SD3.tif]

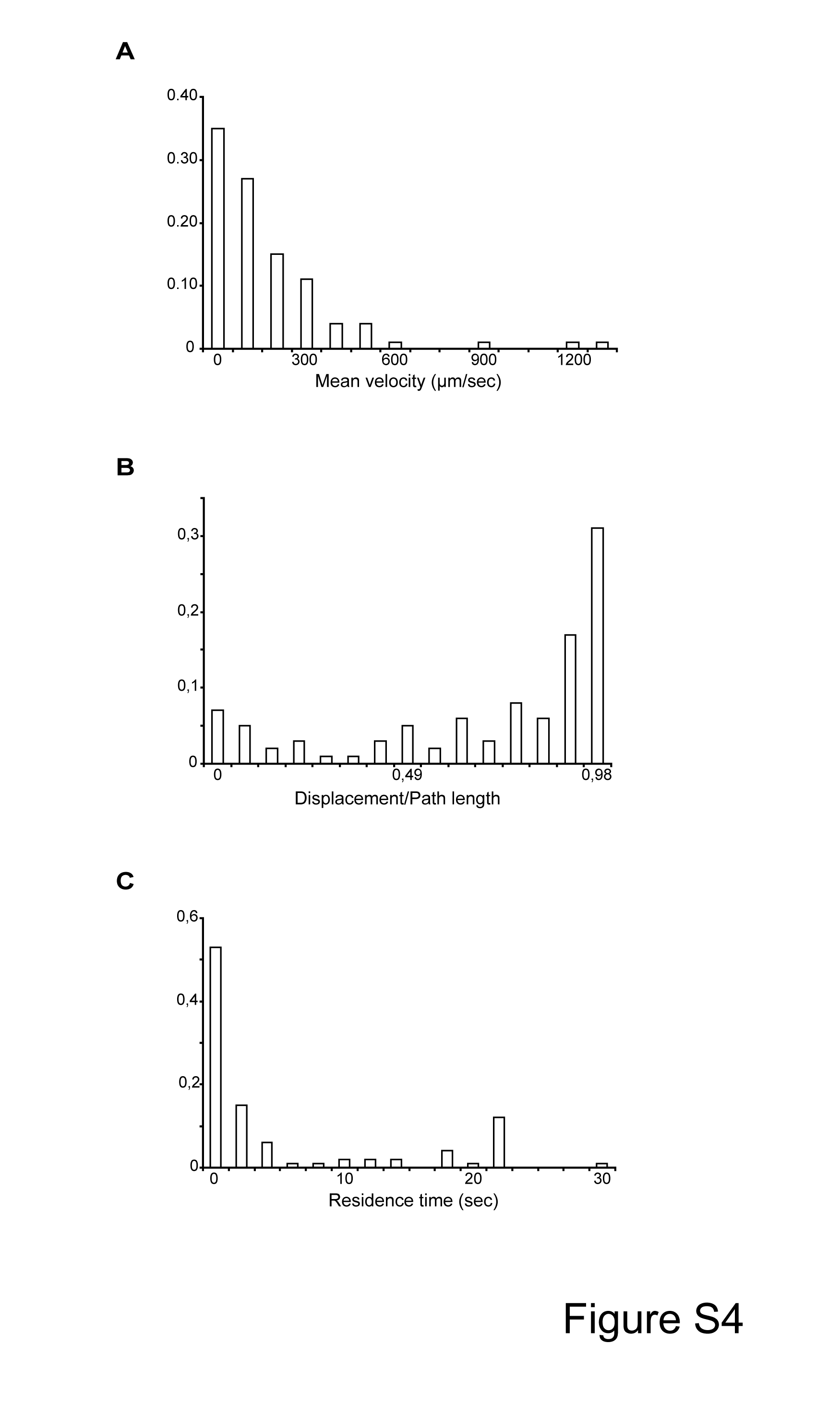

Supplement: Supplementary file 4 [file cmi0013-0109-SD4.tif]

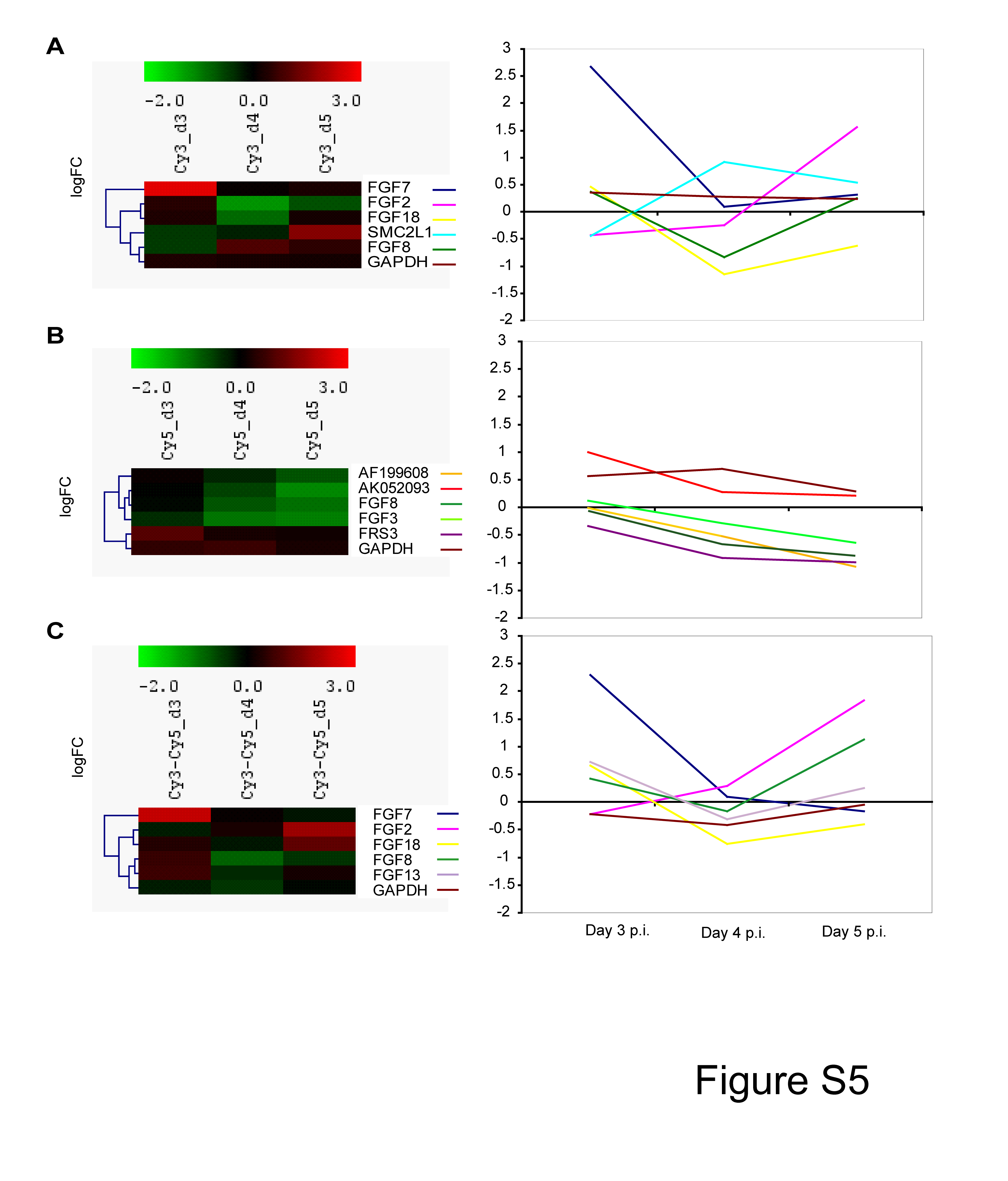

Supplement: Supplementary file 5 [file cmi0013-0109-SD5.tif]

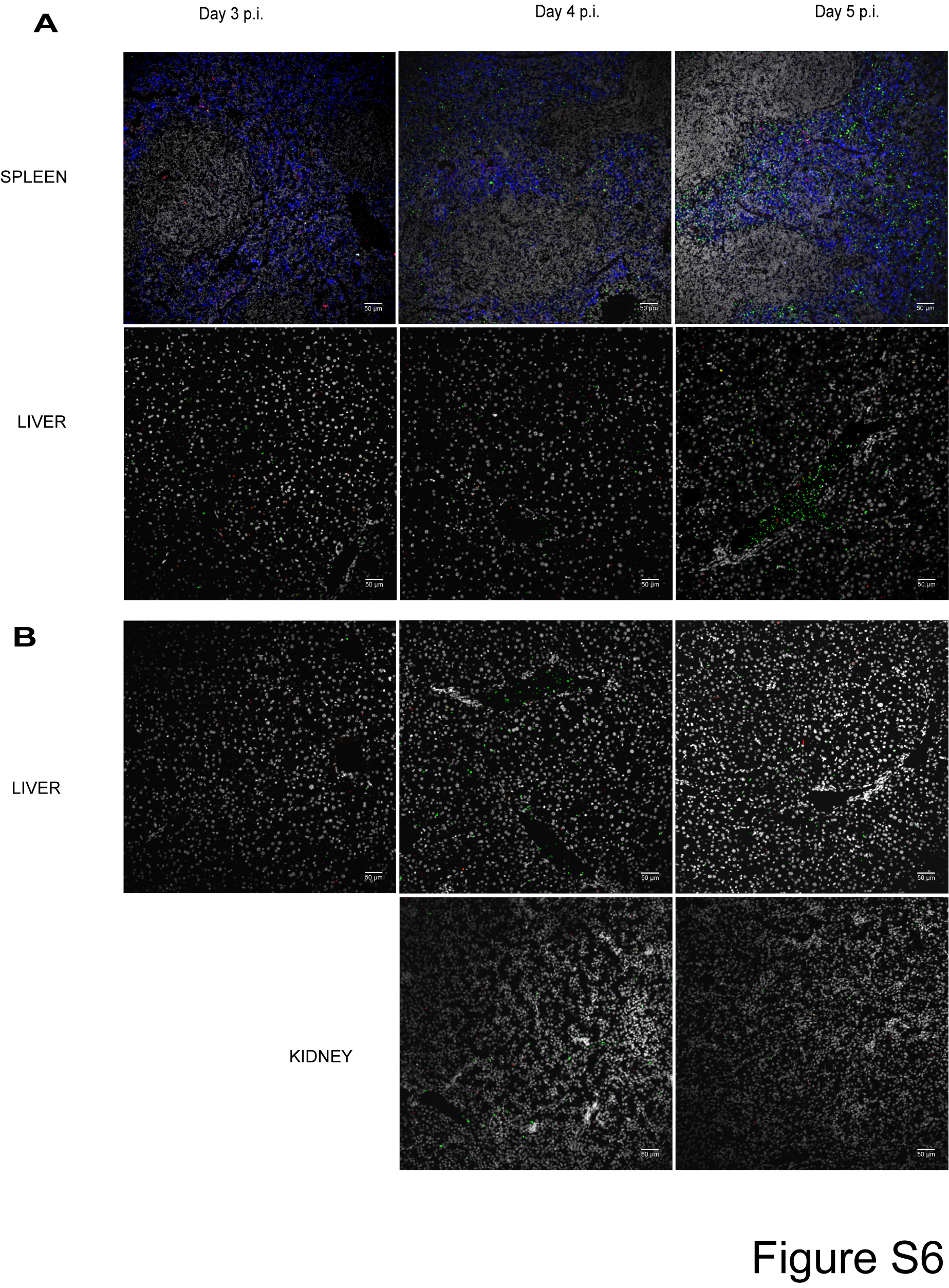

Supplement: Supplementary file 6 [file cmi0013-0109-SD6.tif]

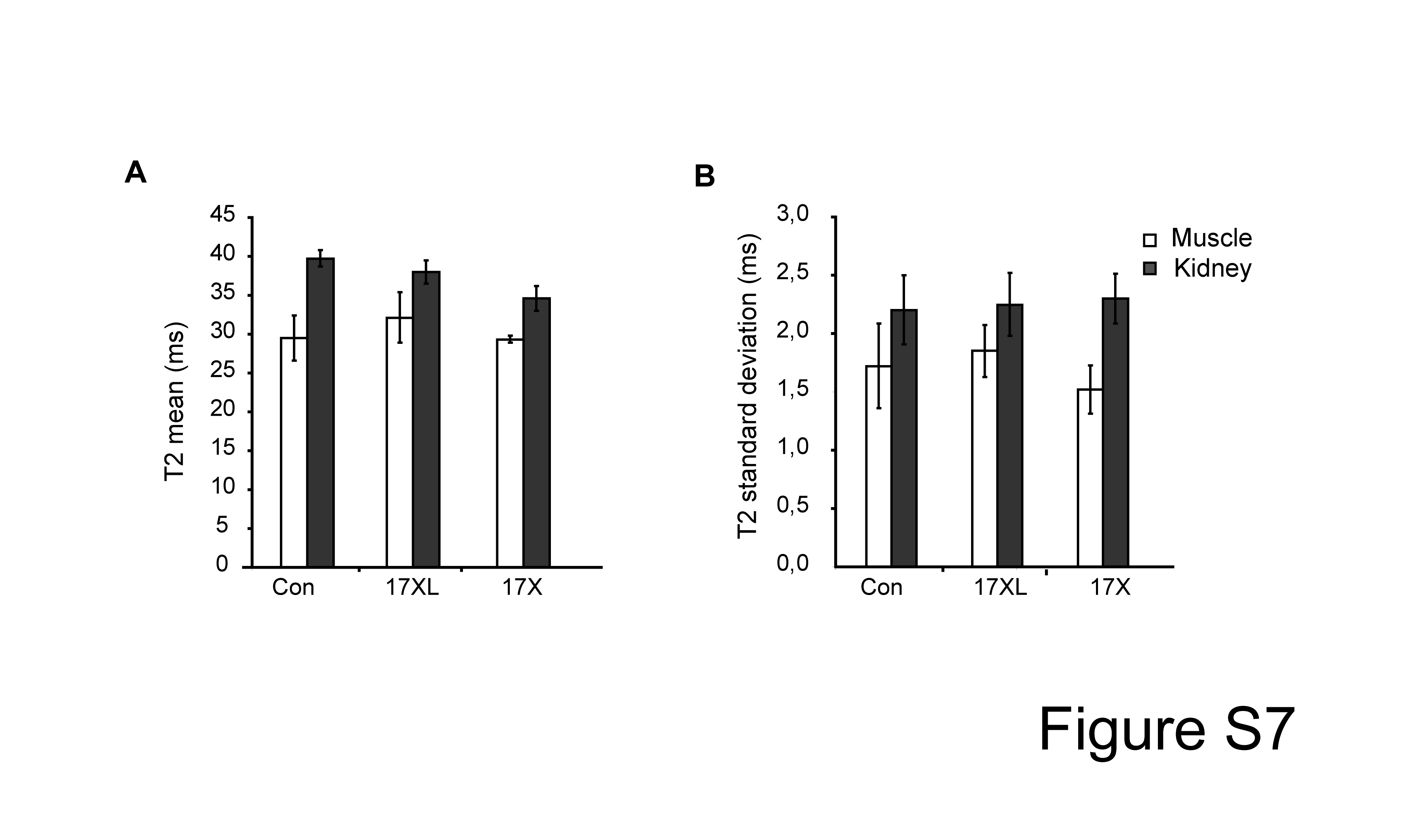

Supplement: Supplementary file 7 [file cmi0013-0109-SD7.tif]
